# Anisotropic Cellulose Nanofibers/Polyvinyl Alcohol/Graphene Aerogels Fabricated by Directional Freeze-drying as Effective Oil Adsorbents

**DOI:** 10.3390/polym11040712

**Published:** 2019-04-18

**Authors:** Lijie Zhou, Shengcheng Zhai, Yiming Chen, Zhaoyang Xu

**Affiliations:** College of Materials Science and Engineering, Nanjing Forestry University, Nanjing 210037, China; zhoulijie678@163.com (L.Z.); zhai_sc@njfu.edu.cn (S.Z.); 18362983199@163.com (Y.C.)

**Keywords:** cellulose nanofibers, polyvinyl alcohol, graphene, directional freeze-drying, oil absorption

## Abstract

Under the current situation of frequent oil spills, the development of green and recyclable high-efficiency oil-absorbing aerogel materials has attracted wide attention from researchers. In this study, we report a high-strength, three-dimensional hydrophobic cellulose nanofiber (CNF)/polyvinyl alcohol (PVA)/graphene oxide (GO) composite aerogel with an anisotropic porous structure, which was fabricated by directional freeze-drying technology using anisotropically grown ice crystals as a template, followed by hydrophobic treatment with a simple dip coating process. The prepared composite aerogel presented anisotropic multi-level pore microstructures, low density (17.95 mg/cm^3^) and high porosity (98.8%), good hydrophobicity (water contact angle of 142°) and great adsorption capacity (oil absorption reaching 96 times its own weight). More importantly, the oriented aerogel had high strength, whose compressive stress at 80% strain reached 0.22 MPa and could bear more than 22,123 times its own weight without deformation. Therefore, the CNF/PVA/GO composite aerogel prepared by a simple and easy-to-operate directional freeze-drying method is a promising absorbent for oil-water separation.

## 1. Introduction

Water pollution caused by oil and chemical spills seriously harms the ecological environment and human health [1,2,3]. Therefore, effectively solving the problem of oil pollution has become an urgent and arduous task. Traditional methods for dealing with petroleum pollution include the following methods: physical and mechanical methods [4], direct combustion methods [5], bioremediation methods [6], chemical treatment methods [7] and dosing materials [8], in which the adsorption of adsorbent materials is simple and environmentally friendly. The oil-absorbing materials reported in the literature can be summarized into three categories according to the nature and source of the materials: (1) porous inorganic minerals such as clay [9], silica [10], perlite [11], fly ash [12], etc. These materials come from a wide range of sources, which have low oil absorption, poor oil holding capacity, and difficulty in recycling; (2) synthetic polymers such as polyurethane [13], polystyrene foam [14]. Although these materials have good oil absorption ability and reusability, they have poor biodegradability; (3) natural plant fibers such as cotton fiber [15], straw [16], etc. These materials are provided with a wide range of sources, low cost and biodegradability but lack a good oil absorption rate. Therefore, novel, environmentally friendly, highly efficient and sustainable materials are urgently needed.

Nanocellulose has a wide range of sources, good biodegradability and outstanding mechanical properties [17]. Cellulose aerogel not only possesses the advantages of low density and high porosity compared with traditional aerogel but also has good biocompatibility that makes it an environmentally friendly adsorbent and has attracted widespread attention [18]. Moreover, hydrophobic cellulose-based aerogels can be prepared by coating metal oxides and hydrophobic polymers. It has been studied to modify cellulose-based aerogels with methyltrimethoxysilane and trimethylchlorosilane to make them hydrophobic and lipophilic [19,20]. Studies have shown that hydrophobically modified cellulose-based aerogels are potential oil-absorbing materials, which are used to remove marine oil spills [21,22].

Despite the excellent properties of cellulose-based aerogels, their low strength and modulus limit their application compared to inorganic polymer materials. The poor compressive strength of oil-absorbing aerogels will cause them to be broken during the oil absorption process, so it is important to increase their strength. To enhance the compressive properties of the aerogel, we prepared cellulose nanofiber (CNF) / polyvinyl alcohol (PVA) / graphene oxide (GO) composite aerogels. The low cost PVA solution has excellent water solubility, biodegradability and biocompatibility. The long polymer chains of PVA lead to high-density hydrogen bonding with CNFs and GO, which can enhance the mechanical properties of the cellulose aerogel [23,24,25,26]. Meanwhile, GO forms a strong interaction with CNFs and PVA through hydrogen bonding due to its excellent mechanical properties and large number of oxygen atoms on the surface. However, most of the aerogels reported have a random internal structure, leading to relatively poor compressive strength. In recent years, orientation techniques have been used to prepare porous ceramic materials and have attracted the attention of researchers as a special method for preparing aerogels [27,28,29]. Lee and Deng reported the fabrication of layered cellulose foams by directional freezing, emphasizing the differences in the microstructure and mechanical properties of microfiber foams [30]. Therefore, in order to further increase compressive strength, designing anisotropic porous structures in aerogels by directional freezing may be a viable solution.

**Figure 1:**
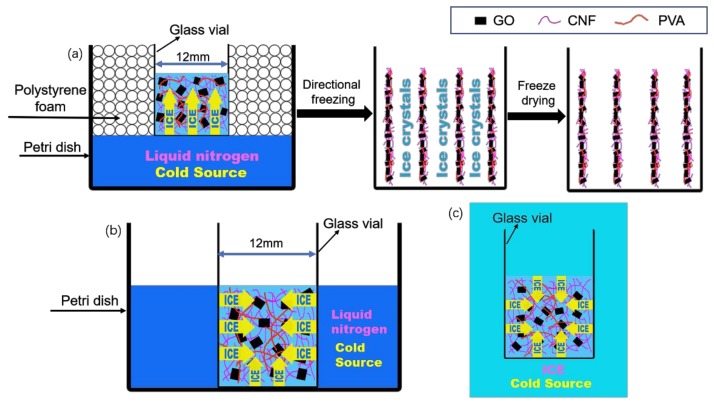
Schematic representation of (**a**) the directional freeze-drying method; (**b**) the non-directional freeze-drying method and (**c**) the refrigerator freeze-drying method. Cellulose nanofiber (CNF); polyvinyl alcohol (PVA); graphene oxide (GO).

“Directional freeze-drying” (Figure 1a) means that the bottom of the container contacts with liquid nitrogen, causing the solvent to freeze from the bottom of the container and that the ice crystals grow in one direction. When the vessel containing the mixed aqueous suspension is frozen, phase separation causes the particles and polymer molecules to be expelled from the formed ice crystals. Then, the molecules are accumulated between the growing ice crystals, and the freezing process can be carried out in a more controlled manner. In this way, a defined alignment structure is formed. After sublimation of the ice crystals after lyophilization, the resulting solids can form a continuous 3D network to form an anisotropic porous aerogel [31]. In addition, containers containing aqueous suspensions are directly frozen in liquid nitrogen and a refrigerator to produce a relatively disordered porous structure (we refer to them as “non-directional freeze-drying” and “refrigerator freeze-drying”) [32,33,34]. “Non-directional freeze-drying” (Figure 1b) means that the bottom of the container and the bottle body are in direct contact with liquid nitrogen, then the ice crystal grows from the outside of the suspension to the inside in the direction of the bottom and the bottle body, followed by lyophilization. “Refrigerator freeze-drying” (Figure 1c) means that when water suspension is frozen by refrigerator, the container is surrounded by cold air, so the ice crystal grows from the outside of the suspension to the inside in all directions. Due to the different growth patterns of ice crystals during freezing, the three composite aerogels prepared by different freezing methods have different structures and properties.

In this study, a novel, simple, safe and reliable "directional freezing" method was used to prepare a CNF/PVA/GO composite aerogel with an anisotropic alignment structure. Then, CPGA was hydrophobically modified with methyltrichlorosilane (TMCS) by simple thermal chemical vapor deposition to obtain a hydrophobic CNF/PVA/GO composite aerogel (MCPGA). To confirm the properties of the resulting aerogel, characterization tests were performed by scanning electron microscopy (SEM), Fourier transform infrared (FTIR) spectroscopy, mechanical testing machine and water contact angle (WCA). The results show that the obtained composite aerogel has a low density, high porosity, aligned porous structure and high compressive strength. It also exhibited high oil-water selectivity and excellent oil absorption capacities for various oils and organic solvents.

## 2. Experimental

### 2.1. Materials

Bamboo powder (60 mesh) was purchased from Chongqing Dongyi Xiabu Co., Ltd. (Chongqing, China) and was the raw material for preparing nanocellulose. Graphite powder was provided by Qingdao Henglide Graphite Co., Ltd. (Qingdao, China). Analytical grade PVA (Mw: 95,000 g/mol), glutaraldehyde (GA, Crosslinker, 25 wt % in H_2_O), potassium hydroxide (KOH), Sudan III, TMCS (99 wt %), acetic acid (CH_3_COOH), hydrogen peroxide (H_2_O_2_, 30%), sodium nitrate (NaNO_3_), potassium permanganate (KMnO_4_), concentrated sulfuric acid (H_2_SO_4_, 98%), phosphorus pentoxide (P_2_O_5_), hydrochloric acid (HCl), sodium chlorite (NaClO_2_) and potassium persulfate (K_2_S_2_O_8_) were purchased from Aladdin Industrial Co (Shanghai, China). All materials were analytical and used without further purification. Deionized water was self-made in our laboratory.

### 2.2. Preparation of Cellulose Nanofibers

The chemical treatment of bamboo powder referred to the treatment method is described in the document [35]. The cellulose suspensions used in this study were prepared according to the methods reported in the previously reported literature [36]. The prepared cellulose suspensions were stored at 4 °C before future utilization.

### 2.3. Preparation of Polyvinyl Alcohol Solution

PVA (10.0 g, Mw: 95,000 g/mol) was dissolved in deionized water (100 mL) and stirred in a water bath (HH-1, Nanjing Xian’ou Instrument Manufacturing Co., Ltd., Nanjing, China) at 85 °C for 12 h until the PVA was completely dissolved. The PVA solution was then stored at room temperature for further use.

### 2.4. Preparation of Graphene Oxide

GO was prepared according to a modified Hummers’ method [37]. Natural graphite powder (3 g), K_2_S_2_O_8_ (4 g) and P_2_O_5_ (4 g) were mixed and placed into a water-bath (80 °C) with concentrated H_2_SO_4_ (25 mL). After treatment for 5 h under strong mixing by magneton, the mixture was washed with distilled water until the solution was neutral, then placed in a drying oven (60 °C) (DZF-6090, Shanghai Jinghong Laboratory Equipment Co., Ltd., Shanghai, China). The mixture, NaNO_3_ (1 g) and KMnO_4_ (10 g) were slowly added into a beaker pre-filled with concentrated H_2_SO_4_ (100 mL) in the ice-bath under strong stirring for 2 h. Then, the mixture was warmed to 35 °C and kept for 4 h, heated to 90 °C with adding distilled water (800 mL) and held for 1 h, and then mixed with H_2_O_2_ (20 mL) to a bright yellow solution. The solution was filtered with HCl (5 wt %) for several times and the undissolved substance was washed with deionized water until it was neutral. In order to remove the residual salts and acids, the resulting graphite oxide colloid was dialyzed with a molecular weight cut-off membrane (MW 3500 Da) and deionized water for 2 weeks at a room temperature. Finally, the solution was diluted to the concentration of 1.5 wt %; then, exfoliation of graphite oxide to graphene oxide sheets was performed by sonicating for 0.5 h under the condition of 960 W, and a homogeneous GO solution was obtained.

### 2.5. Preparation of CNF/PVA/GO (CPGA) by Directional Freeze-drying

The CNF suspension (3 g, 1.275 wt %), PVA solution (0.76 mL, 0.1 g/mL), GO solution (3.825 g, 1.5 wt %) and water (2 mL) were mixed and vigorously stirred in a beaker for 1 h. Sulfuric acid (8 μL, 1.0% by volume) was added to the above suspension to adjust the pH of the mixture from 4 to 6. Then, the GA solution (80 μL, 25 wt %) was added to the resulting CNF/PVA/GO mixture. The above mixture was mechanically stirred for 1 h and sonicated in an ultrasonic bath for 30 min, and finally placed in a vacuum oven to remove any residual air bubbles. At the final stage, the obtained mixture was transferred to a cylindrical mold and then crosslinked in an oven at 75 °C for 3 h. The crosslinked aqueous gel was stored overnight in a 4 °C refrigerator for pre-cooling to avoid macroscopic cracking during the freezing step. A cylindrical mold with a mixed aqueous gel was placed in a directional freezer for a few minutes to ensure it was completely frozen; then, the mold was transferred to a freeze dryer and lyophilized for 48 h at −50 °C, and then the directional CNF/PVA/GO aerogel (d-CPGA) was obtained. In addition, refrigerator freeze-dried CNF/PVA/GO aerogel (r-CPGA) and non-directional freeze-dried CNF/PVA/GO aerogel (n-CPGA) were prepared for comparison.

### 2.6. Modification of CPGA to Prepare Hydrophobic CPGA Aerogel (MCPGA)

**Figure 2:**
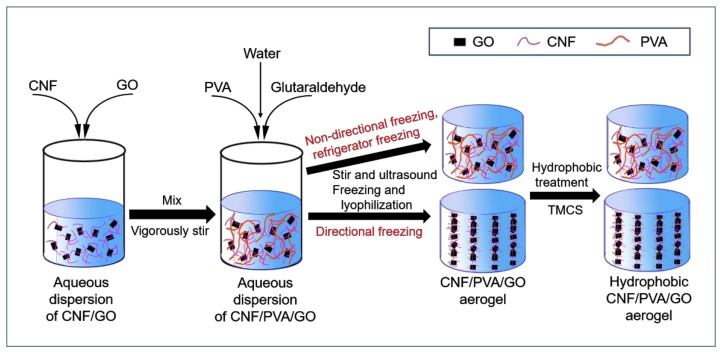
Schematic showing experimental process of the samples.

In order to prepare the hydrophobic composite aerogel, TMCS was used as a modification agent. The CPGA (d-CPGA, r-CPGA, n-CPGA) was placed in a sealed container together with 8 mL of TMCS in an oven at 40 °C for 10 h to produce a hydrophobic and oleophilic MCPGA (d-MCPGA, r-MCPGA, n-MCPGA) by a silanization reaction. All experimental steps of the sample were shown in Figure 2.

### 2.7. Density and Porosity Measurements

The density (ρ) of aerogels was calculated according to Equation (1):

(1) ρ=m/v

where ρ is the density of the aerogel, m is the mass of the aerogel, and v is the volume of the aerogel.

The density ($\rho_{s}$) of the solid material is calculated according to Equation (2) based on the solid density of each component and their weight ratios:

(2) $$\rho_{s}=1/\left(W_{CNFs}/\rho_{CNFs}+W_{PVA}/\rho_{PVA}+W_{GO}/\rho_{GO}+W_{silane}/\rho_{silane}\right)$$

where W is the weight percentage of the different components. $\rho_{CNFs}$, $\rho_{PVA}$, $\rho_{GO}$ and $\rho_{silane}$ are the solid densities of CNFs, PVA, GO and silane, respectively. The densities of the CNFs, PVA, GO and silane used for this study are 1460, 1269, 2100 and 1273 kg/m^3^, respectively, according to the manufacturer’s data sheet.

The porosity (P) of aerogels was calculated according to Equation (3)

(3) $$P\left(\%\right)=\left(1-\rho /\rho_{S}\right)\times 100\%$$

where P, ρ and $\rho_{s}$ are the porosity, actual density and relative density of the material, respectively.

### 2.8. Sample Characterization

For each test described below, each sample was tested at least three times and the average results were reported. Field emission scanning electron microscopy (FE-SEM, Hitachi S-4800, Tokyo, Japan) was used to investigate the microscopic morphology of different types of aerogels. The Fourier transform infrared spectroscopy (FTIR) (Nicolet iS10, Thermo Electron Corp., Madison, WI, USA) data was recorded in the range of 500–4000 cm^−1^. X-ray diffraction (XRD) pattern was measured on Ultima IV multipurpose XRD system (Ultima IV, Rigaku, Akishima, Japan) with Cu Kα radiation at a scanning rate of 5°/min. The water contact angle was measured at room temperature using a contact angle goniometer (OCA 15/20, Future Digital Scientific Corp., Natick, MA, USA) with 4 μL of water droplets. The static drop method (angle measurement method) was used for WCA testing. Three aerogels of the same type were taken and the droplets were separately dropped onto the surface of the aerogel for 2 min with deionized water. The data was read at 1 min and 2 min and the arithmetic mean was taken, respectively. The data was obtained after multiple measurements. The compression test was carried out at room temperature using Shimadzu CMT4204 (Kyoto, Japan). The test sample had a diameter of 12 mm and a height of 8 mm and the loading rate was set to 2 mm/min. The oil absorption capacity of MCPGA, Qt, was measured using the equation Qt  =  (m_2_ − m_1_)/m_1_, where m_1_ and m_2_ are the weights of the aerogels before and after absorption. The oil absorption capacity is the average of all absorption tests repeated three times.

## 3. Results and Discussion

### 3.1. Morphology and Microstructure

**Figure 3:**
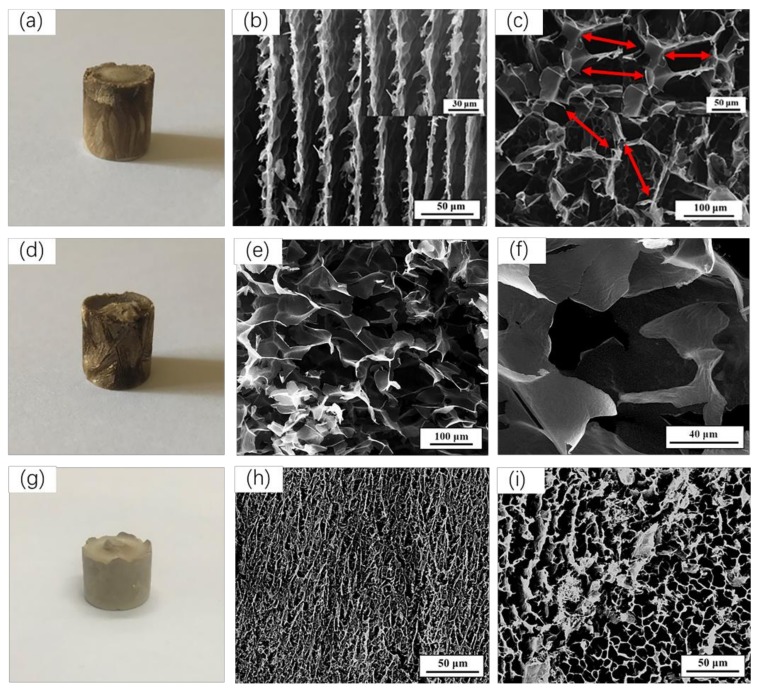
Macroscopic pictures and microstructures of aerogels formed by different freeze-drying methods. (**a**), (**d**) and (**g**) are macro photographs of d-MCPGA, r-MCPGA, n-MCPGA, respectively; (**b**) vertical section of d-MCPGA; (**c**) cross section of d-MCPGA; (**e**–**f**) internal SEM image of r-MCPGA; (**h**) vertical section of n-MCPGA; (**i**) cross section of n-MCPGA.

Figure 3a,d,g are digital photographs of d-MCPGA, r-MCPGA, n-MCPGA, respectively. From the pictures, we could not find the difference in macroscopic appearance. In order to explore the difference in the internal structure of the aerogel, we observed the microstructure by SEM (Figure 3b–f,h–i). As shown in the c, e and i of Figure 3, d-MCPGA, r-MCPGA, and n-MCPGA all had a three-dimensional interpenetrating porous structure. Previous studies have shown that solidification has an important influence on the microstructure and pore morphology of porous materials [38,39]. D-MCPGA produces an aligned layered structure in the vertical direction. This particular structure strongly indicates that the ice crystals grow mainly in the vertical direction, and the sheet fibers of d-MCPGA grow parallel to the freezing direction, which indicates the d-MCPGA had anisotropic porous structure and the pore size was about 60 μm (Figure 3b–c). Furthermore, as indicated by the red markings in Figure 3c, there were a plurality of connecting channels between the holes which formed an open porous structure that allowed the oil to easily reach the interior of the aerogel to enhance oil absorption. Freezing at −40 °C had the slowest freezing rate and produced the largest ice crystals, so r-MCPGA had a maximum pore size of nearly 70 μm and a disordered three-dimensional porous structure (Figure 3e–f). For n-MCPGA, the freezing rate of the frozen slurry in liquid nitrogen was too fast to produce a dense structure with only a small pore size of 10 μm (Figure 3i). Under such rapid freezing conditions, the thin wall was thin and the space between the sheets was small, which was not conducive to the entry of oil and organic solvents.

The MCPGA were ultra-light and had high porosity. The density and porosity of MCPGA (d-MCPGA, r-MCPGA, n-MCPGA) are summarized in Table 1.

### 3.2. Chemical Properties

**Figure 4:**
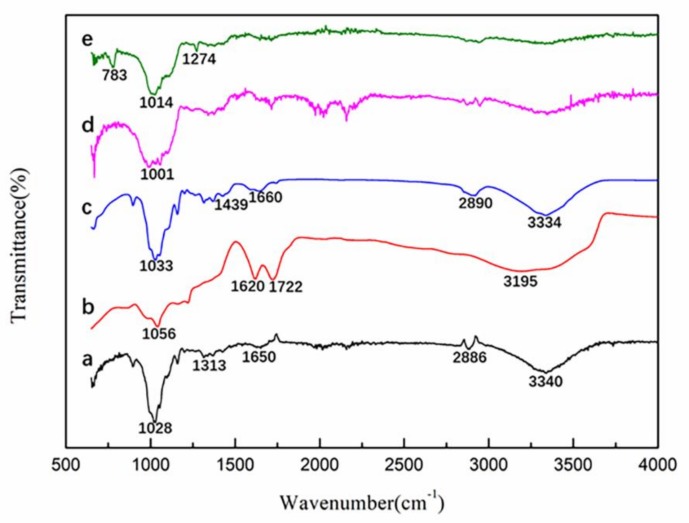
FTIR spectra of (**a**) CNFs, (**b**) GO, (**c**) PVA, (**d**) CNF/PVA/GO and (**e**) TMCS /CNF/PVA/GO aerogels.

To evaluate the formation of TMCS/CNF/PVA/GO composites, we used Fourier transform infrared spectrometer and an X-Ray diffractomer. Figure 4 showed FTIR spectra of (a) CNFs, (b) GO, (c) PVA, (d) CNF/PVA/GO and (e) TMCS/CNF/PVA/GO aerogels.

The spectrum of CNFs (Figure 4a) showed that 3340 cm^−1^ and 1650 cm^−1^ were the stretching vibration and bending vibration of -OH, 2886 cm^−1^ was the stretching vibration of CH, 1313 cm^−1^ was the bending vibration of -OH, 1028 cm^−1^ was the stretching vibration absorption peak of C-O, whose vicinity had many weak shoulder peaks [35,40]. The spectrum of GO (Figure 4b) showed a strong peak corresponding to an oxygen-containing group. A broad peak near 3195 cm^−1^ was a hydroxyl group, 1722 cm^−1^ was a C=O stretching vibration from a carbonyl group and a carboxyl group, 1620 cm^−1^ was C=C from an aromatic ring, 1225 and 1056 cm^−1^ were C-O-C and C-O stretching vibrations, respectively [41]. In the infrared spectrum of polyvinyl alcohol (Figure 4c), 3334 cm^−1^ and 2890 cm^−1^ were stretching vibrations of -OH and -CH_2_, respectively [42], 1660 cm^-1^ was H-O-H bending vibration, 1439 cm^−1^ was CH stretching vibration and 1033 cm^−1^ was the C-O-C stretching vibration absorption peak [43]. In the CNF/PVA/GO spectrum (Figure 4d), we could find that the diffraction peaks of CNFs, PVA, and GO all appeared, but the peaks were weakened, which might indicate that a strong interaction was forced among the three components of CNFs, PVA, and GO [34]. Figure 4e was the FTIR spectrum of the TMCS/CNF/PVA/GO aerogel. There was no significant change in the characteristic peak of the composite aerogel before and after the TMCS hydrophobic treatment. However, new diffraction peaks appeared at 783 and 1274 cm^−1^, which were ascribed to characteristic vibrations of Si-O-Si and C-Si asymmetric stretching, respectively. In the region of 1000–1130 cm^−1^, the Si-O-Si bond absorption band in the siloxane compound overlaps with the C-O bond of cellulose [28], which indicated that the silylation reaction proceeded successfully and only reacted on the surface of the aerogel so that the skeletal structure and mechanical properties of the aerogel were not affected.

**Figure 5:**
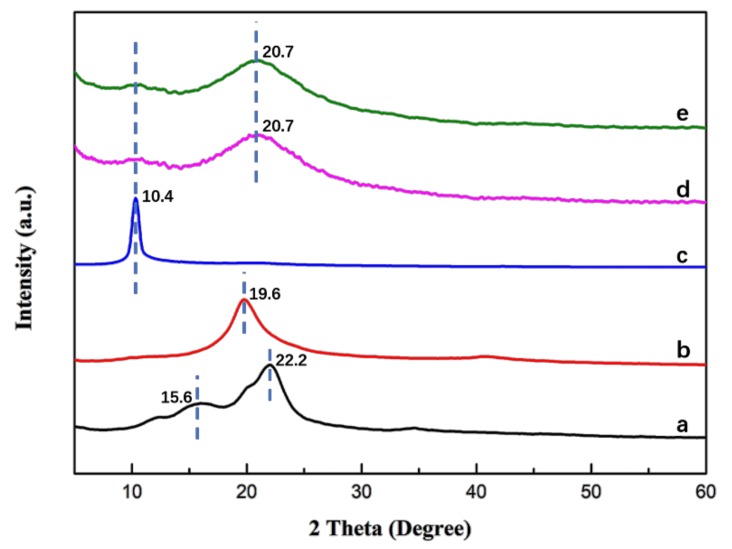
XRD patterns of (**a**) CNFs, (**b**) PVA, (**c**) GO, (**d**) CNF/PVA/GO and (**e**) TMCS/CNF/PVA/GO aerogels.

The XRD patterns of (a) CNFs, (b) PVA, (c) GO, (d) CNF/PVA/GO and (e) TMCS/CNF/PVA/GO aerogels are shown in Figure 5. The distinct peak at 2θ = 22.5° observed from CNFs (Figure 5a) was attributed to the typical reflection planes (002), and a broad peak at around 15.6° was assigned to the (101) and (10$\bar{1}$) lattice planes of the cellulose I crystalline structure [44]. The XRD pattern of the PVA (Figure 5b) exhibited a broad peak at 2θ = 19.6°, which was ascribed to the orthorhombic lattice structure of semi-crystalline PVA [45]. GO exhibited a diffraction peak at 2θ = 10.4° (Figure 5c), corresponding to the (002) crystal plane [46]. The diffraction peaks of the CNFs, PVA and GO all appeared in the CNF/PVA/GO aerogel (Figure 5d). A broad peak was observed at 2θ = 20.7° and the peak intensity decreased, corresponding to the diffraction peaks of the CNFs and PVA. Moreover, the peak of GO sharply weakened, probably due to the relatively small amount of GO added to the sample. The TMCS/CNF/PVA/GO aerogel (Figure 5e) did not show any different diffraction peaks of CNF/PVA/GO aerogel as the TMCS silylation reaction only occurred on the aerogel surface without changing its chemical structure.

### 3.3. Mechanical Properties

**Figure 6:**
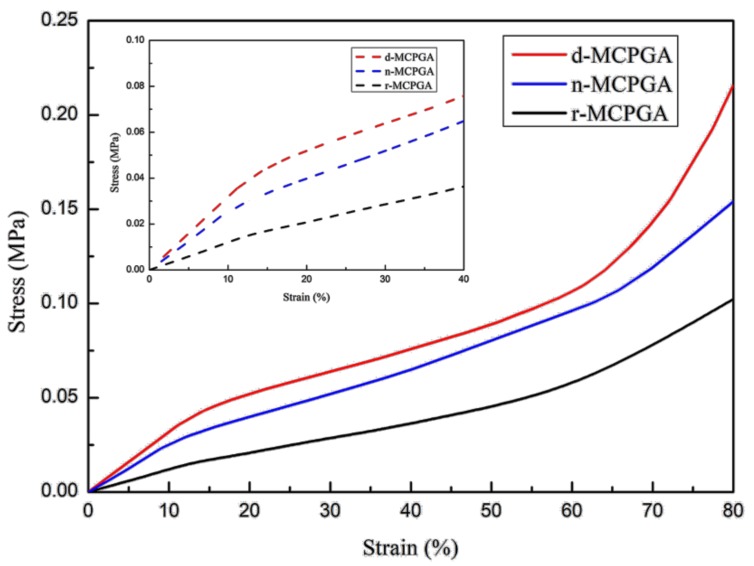
Compressive behavior of aerogels (d-MCPGA, r-MCPGA, n-MCPGA) prepared by different freeze-drying methods consisting of CNFs, PVA and GO.

**Figure 7:**
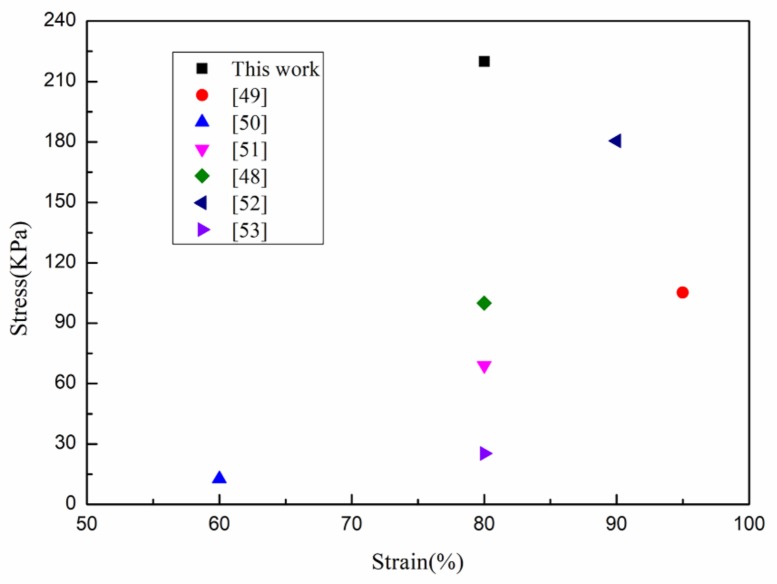
Comparison of the compressive strength of different materials [49,50,51,52,53].

The mechanical properties of aerogels were influenced by many factors, such as raw materials and ratios, material properties of each component, and microstructure. Under the same raw materials and ratios, different preparation methods have produced aerogels with different microstructures. This structural difference played a key role in influencing the mechanical properties of aerogels. The compression behavior of aerogels (d-MCPGA, r-MCPGA, n-MCPGA) made by CNFs, PVA and GO using the directional freeze-drying method is shown in Figure 6, and the compression behavior of aerogels prepared by conventional refrigerator freeze-drying and non-directional freeze-drying are used as contrast. The curve characteristics of this three-characteristic deformation region of Figure 6 are consistent with previous studies [47]. The compressive stress of r-MCPGA at 80% strain reached 0.11 MPa, slightly higher than other cellulose-based aerogels described in previous studies [48]. This result might be attributed to the strong interaction between the CNFs and GO through hydrogen bonding and the addition of PVA with a long polymeric chain that could bond with the high density hydrogen of the CNFs and GO. At 80% strain, the ultimate compressive stress of d-MCPGA (0.22 MPa) was two times higher than that of r-MCPGA (0.11 MPa) and about 1.5 times higher than that of n-MCPGA (0.15 MPa). This significant increase might be due to the microstructure of the radially aligned directional aerogel (Figure 3b). Moreover, the d-MCPGA had a higher compressive capacity than most of the reported oil-absorbing materials. Figure 7 compares the compressive strength of d-MCPGA and other oil-absorbing materials. The directional freezing process caused the aerogel to have an axially ordered vertical pore structure, which contributed to the high strength characteristics of the d-MCPGA, but the extrusion behavior destroyed its internal orientation structure and caused the structure to be unrecoverable; thus, d-MCPGA had high strength but was relatively inferior in flexibility and repeatability.

**Figure 8:**
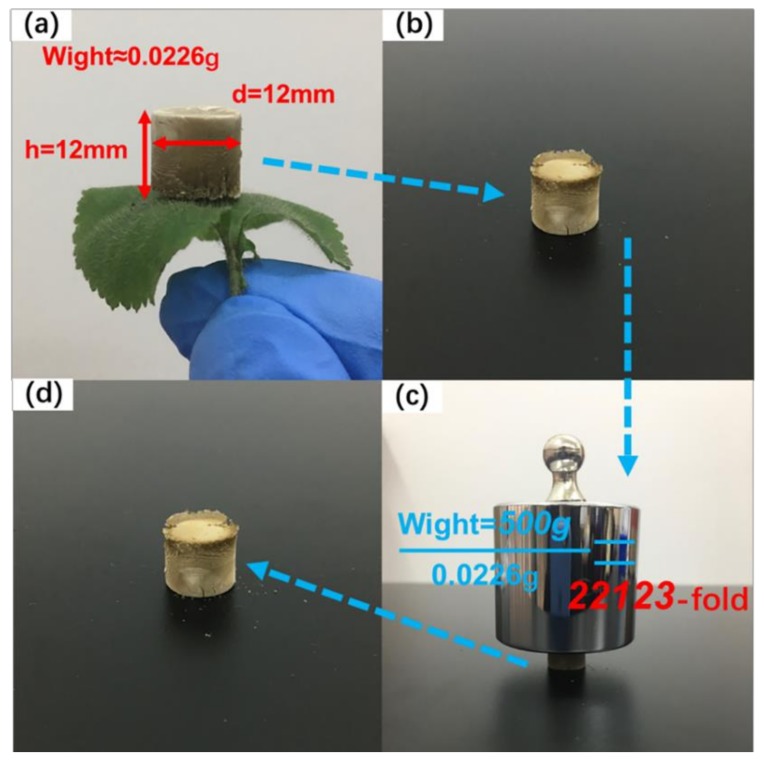
(**a**) Ultra-light cylindrical d-MCPGA supported by young leaves; (**b**–**d**) the pre, middle and post processes of the d-MCPGA supporting a 500 g mass load.

As shown in Figure 8a–d, 0.0226 g of lightweight d-MCPGA were placed on the young leaves and carried a weight of 500 g without any deformation (bearing more than 22123 times its own weight), which far exceeded the previous the study. This high strength was mainly attributed to the excellent entanglement of CNFs with PVA and GO, and the directional freeze-drying method formed a highly oriented microstructure.

### 3.4. Surface Wettability

**Figure 9:**
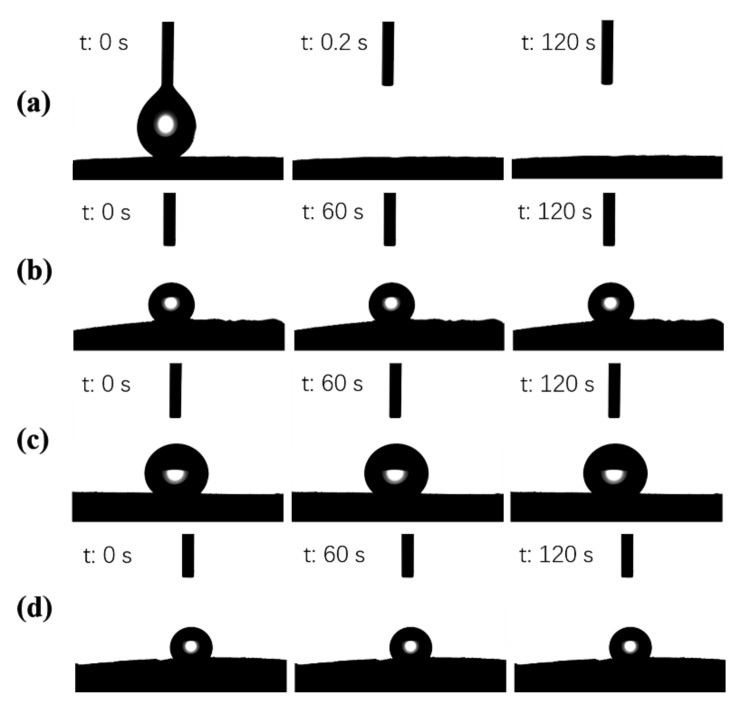
Water contact angle of (**a**) CPGA, (**b**) d-MCPGA, (**c**) r-MCPGA and (**d**) n-MCPGA.

Hydrophobicity was one of the most important criteria for evaluating oil-absorbing materials. The surface wettability of CPGA, MCPGA (d-MCPGA, r-MCPGA, n-MCPGA) was investigated by contact angle measurement. Due to the presence of abundant hydroxyl groups hydroxyl groups on the surface of the CNFs, PVA and GO, CPGA exhibited strong hydrophilicity. As shown in Figure 9a, for the original CPGA prior to silane coating, 4 μL of water droplets were completely absorbed by in 0.2 s and the WCA of 0°. However, the silane-coated d-MCPGA, r-MCPGA, and n-MCPGA were all hydrophobic; the WCA was as high as 142, 138 and 139 (Figure 9b–d). After 120 s, the water droplets retained the initial contact angle and were not absorbed by the aerogel. The water contact angle of the outer surface did not change significantly with time. Various MCPGAs had a microporous structure, which could promote the complete filling of high porosity aerogels by TMCS. These results indicate that the aerogel had a highly hydrophobic surface after silane treatment.

### 3.5. Absorption Capacity

**Figure 10:**
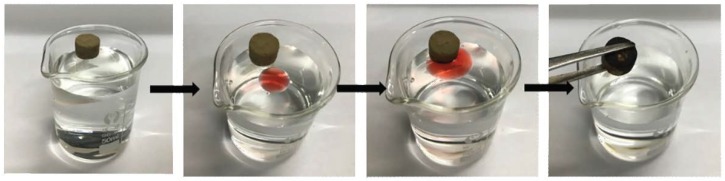
Removal of corn oil (dyed with Sudan red) from the water surface using MCPGA.

**Figure 11:**
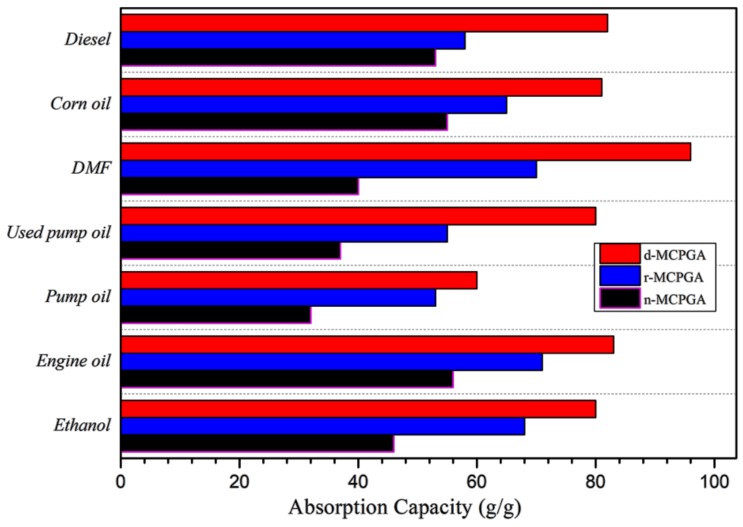
Absorption capacity of MCPGA (d-MCPGA, r-MCPGA, n-MCPGA) for different oils and organic liquids.

Silane-coated hydrophobic lipophilic MCPGA was an ideal absorbent material for the removal of oils and organic solvents from water. Due to its porous structure and oil-water selectivity, MCPGA has a strong ability to selectively absorb oil and organic liquids in water. Figure 10 showes the process of MCPGA removal of corn oil (stained with Sudan Red). MCPGA and dyed corn oil didn’t touch and float on the surface of deionized water. When the two substances were in contact, the oil was quickly absorbed by MCPGA. After removing MCPGA, the remaining was clean water without any oil. In order to further study the oil absorption capacity of the MCPGA, we measured the adsorption capacity of different oils and organic solvents by weighing the samples before and after oil absorption. Figure 11 showed the absorption capacity of the MCPGA (d-MCPGA, r-MCPGA, n-MCPGA) for different oils and organic liquids. The oils and organic liquids tested included diesel, corn oil, N,N-dimethylformamide, pump oil, secondary pump oil, engine oil and ethanol.

The absorption ratios of d-MCPGA, r-MCPGA and n-MCPGA were different. The oil absorption capacity of d-MCPGA was higher (60~96 times), the oil absorption capacity of r-MCPGA was second (53~71 times), while n-MCPGA had the worst oil absorption capacity (32~56 times). The oil was mainly stored in the macropores of the aerogel, so the difference in the absorption capacity between the various organic solvents depended on the surface tension and density of the solvent tested, and the different porous structures and porosities inside the three aerogels. Capillary forces were responsible for the adsorption capacity of aerogels. Due to the capillary interaction between the mobile liquid and the aerogel surface, the liquid could flow spontaneously into the narrow network channel inside the aerogel [54]. Since MCPCA was a porous structure, it had massive capillary channels. Compared to n-MCPGA and r-MCPGA, organic solvents could more easily enter the pores of d-MCPGA through capillary tension. The results of the absorption ratios were consistent with the microstructure and porosity results of the three aerogels previously analyzed. In addition, the oil absorption capacity of d-MCPGA was higher than most existing oil absorption materials (Table 2).

**Figure 12:**
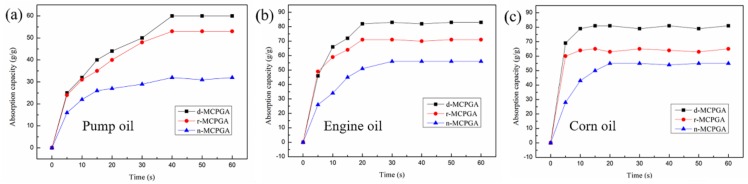
The absorption capacity of MCPGA (d-MCPGA, r-MCPGA, n-MCPGA) to adsorb (**a**) pump oil; (**b**) engine oil and (**c**) corn oil as a function of time.

As shown in Figure 12, the absorption capacity of (a) pump oil; (b) engine oil and (c) corn oil on the MCPGA (d-MCPGA, r-MCPGA, n-MCPGA) was plotted as a function of absorption time. MCPGA took about 20–40 s to achieve the absorption balance of oil and pump oil, while in corn oil it only needed 10 s. The greater the viscosity of liquid, the worse the fluidity and the slower the movement of the molecules. Therefore, the pump oil with higher viscosity reached the absorption saturation in a longer time (viscosity: pump oil > engine oil > corn oil). During the adsorption process, initial adsorption rate was faster and slowed down over time until the adsorption stopped, which might be attributed to the pore structure of the porous aerogel providing a large number of channels for the oil. In addition, the surface and interior of the aerogel possessed a large number of available active adsorption sites, which tended to saturate with time [36]. There was no significant difference in the adsorption rate of d-MCPGA and r-MCPGA. As can be seen from Figure 12b,c, the adsorption rate of n-MCPGA was relatively slow, which might be due to its smaller pores and the narrower channels.

## 4. Conclusions

In summary, we have successfully fabricated hydrophobic anisotropic CNF/PVA/GO aerogel for oil adsorbents using a simple directional freeze-drying process followed by thermal chemical vapor deposition of TMCS. The CNF/PVA/GO aerogel provided a parallel wall porous structure, thereby achieving an excellent oil and organic solvent absorption capacity of up to 96 times its own weight. Further, the assembled anisotropic aerogel delivered low density (17.95 mg/cm^3^), high strength (the compressive stress at 80% strain reaching 0.22 MPa and bearing capacity exceeds 22,123 times its own weight). Therefore, this high strength MCPGA is a potential selective oil absorption material.

## Figures and Tables

**Table 1:** Physical properties of MCPGA (d-MCPGA, r-MCPGA, n-MCPGA).

Sample	Density (kg/m^3^)	Porosity (%)
d-MCPGA	17.95	98.8
r- MCPGA	15.41	99.0
n-MCPGA	18.04	98.8

**Table 2:** Comparison of the absorption capacities of different materials.

Absorbent Material	WCA	Absorption Capacity (g/g)	Ref.
TMCS/rGO/CNF aerogel	117	33–39	[36]
TiO_2_-coated nanocellulose aerogel	>90	40	[55]
Spongy graphene	95	20–86	[56]
Graphene-based aerogel	>90	28–40	[57]
MTMS-coated cellulose aerogel	135	18–20	[58]
CNF aerogel chitosan-silica aerogel	Unknown 137	28 13–30	[59] [60]
Carbon fiber aerogel from bamboo	145	22–80	[61]
Kymene-coated CNF aerogel	144	24–46	[62]
Anisotropic CNF/PVA/GO aerogel	142	60–96	This work
